# Terminal Complement Activation Is Induced by Factors Released from Endplate Tissue of Disc Degeneration Patients and Stimulates Expression of Catabolic Enzymes in Annulus Fibrosus Cells

**DOI:** 10.3390/cells12060887

**Published:** 2023-03-13

**Authors:** Amelie Kuhn, Jana Riegger, Graciosa Q. Teixeira, Markus Huber-Lang, John D. Lambris, Cornelia Neidlinger-Wilke, Rolf E. Brenner

**Affiliations:** 1 Division for Biochemistry of Joint and Connective Tissue Diseases, Department of Orthopedics, Ulm University, 89081 Ulm, Germany; 2 Institute of Orthopedic Research and Biomechanics, Trauma Research Centre, Ulm University, 89081 Ulm, Germany; 3 Institute of Clinical and Experimental Trauma Immunology, University Hospital Ulm, 89081 Ulm, Germany; 4 Department of Pathology and Laboratory Medicine, Perelman School of Medicine, University of Pennsylvania, Philadelphia, PA 19104, USA

**Keywords:** innate immunity, inflammation, complement system activation, disc degeneration, terminal complement complex, complement inhibition

## Abstract

Terminal complement complex (TCC) deposition was identified in human degenerated discs. To clarify the role of terminal complement activation in disc degeneration (DD), we investigated respective activating mechanisms and cellular effects in annulus fibrosus (AF) cells. Isolated cells from human AF, nucleus pulposus (NP), and endplate (EP) were stimulated with human serum alone or with zymosan and treated with either the C3 inhibitor Cp40 or the C5 antibody eculizumab. Complement activation was determined via anaphylatoxin generation and TCC deposition detection. Thereby, induced catabolic effects were evaluated in cultured AF cells. Moreover, C5 cleavage under degenerative conditions in the presence of AF cells was assessed. Zymosan-induced anaphylatoxin generation and TCC deposition was significantly suppressed by both complement inhibitors. Zymosan induced gene expression of ADAMTS4, MMP1, and COX2. Whereas the C3 blockade attenuated the expression of ADAMTS4, the C5 blockade reduced the expression of ADAMTS4, MMP1, and COX2. Direct C5 cleavage was significantly enhanced by EP conditioned medium from DD patients and CTSD. These results indicate that terminal complement activation might be functionally involved in the progression of DD. Moreover, we found evidence that soluble factors secreted by degenerated EP tissue can mediate direct C5 cleavage, thereby contributing to complement activation in degenerated discs.

## 1. Introduction

Disc degeneration (DD) is one of the major causes of lower back pain, a widespread health issue of significant economic relevance [1,2]. DD is associated with structural changes, including lamellar disorganization and fissures, cell-cluster formation, imbalanced nutrition of the tissue, and reduced disc height as well as loss of hydration due to decreased proteoglycan production [3,4,5,6,7]. Furthermore, DD is characterized by metabolic dysregulation and the enhanced production of matrix-degrading enzymes, such as matrix metalloproteinases (MMPs), A disintegrin and metalloproteinase with thrombospondin motifs (ADAMTS), and the lysosomal protease cathepsin D (CTSD) [3,8,9]. Increased production of cytokines, e.g., tumor necrosis factor (TNF), interleukin (IL)-1⍺/ß, IL-6, and IL-17, as well as the cyclooxygenase (COX)2, promote degenerative and inflammatory responses [3,8,10,11]. Under physiological conditions, the intervertebral disc (IVD) is described as immune-privileged due to the avascularity of the tissue [12]. However, due to DD progression, vascularization enables the infiltration of immune cells such as neutrophils, macrophages, or T cells, and it reinforces the increase of pro-inflammatory cytokines [10,13].

**Figure 1:**
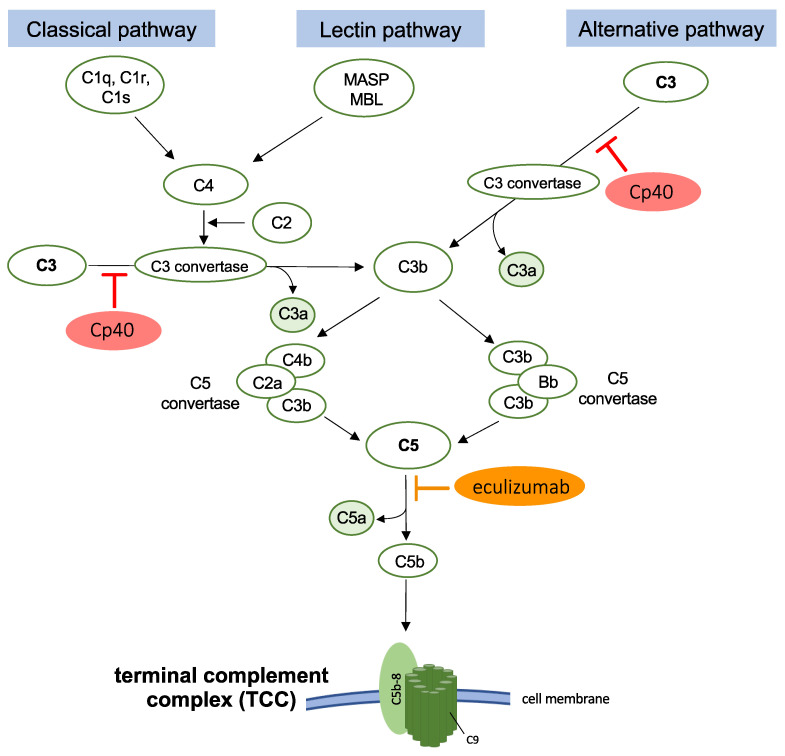
Schematic illustration of the complement cascade. Complement activation conventionally occurs via three main pathways (classical pathway, lectin pathway, or alternative pathway) and starts a cascade that leads to activation of the central factor C3. C3 convertase mediates the cleavage of C3 into anaphylatoxin C3a and C3b. Subsequent cleavage of C5 into anaphylatoxin C5a and C5b is mediated by C5 convertase (C4bC2aC3b or C3bBbC3b). C5b represents the initial component of terminal complement complex (TCC) assembly, which is formed by C5b, C6, C7, C8, and C9 molecules at a cell membrane. C3 inhibitor Cp40 prevents the cleavage of the central complement component C3. Eculizumab inhibits the cleavage of C5. Both inhibitors thereby prevent activation of the terminal pathway (TCC assembly).

The complement system, as part of the innate immune system, can mediate an inflammatory response, damage clearance, and cell death. Thereby, the complement system can be activated via three main pathways or via direct cleavage of C5. Complement activation triggers a cascade of complement factors that leads to the generation of anaphylatoxins C3a and/or C5a as well as the opsonization of damaged or infected tissue by C3b. C3 (C4b2a, C3(H_2_O)Bb, and C3bBb) and C5 (C4bC2aC3b or C3bBbC3b) convertases thereby catalyze the cleavage of C3 and C5, respectively [14]. Cleavage of C5 finally leads to the assembly of C5b, C6, C7, C8, and several C9 molecules, forming the terminal complement complex (TCC, C5b-9; Figure 1), also known as the membrane attack complex (MAC) [15]. In its principal function, the pore-forming TCC mediates pathogen cell lysis, whereas sublytic TCC deposition on nucleated cells, such as neutrophiles, macrophages, or others, is known as a pro-inflammatory trigger [16]. Previously, Grönblad et al. reported that degenerated discs exhibit increased TCC deposition as compared to non-degenerated discs [17]. Furthermore, we showed that the deposition of TCC positively correlates with the grade of degeneration in IVD tissue [18]. Although we observed enhanced levels of the membrane-bound TCC regulator CD59 in human IVD cells due to in vitro expansion, IVD cells could not fully protect themselves against TCC deposition [19]. In osteoarthritis (OA), complement activation, including anaphylatoxin generation and TCC deposition, plays a crucial role in the pathogenesis of the degenerative joint disease [20,21]. Mechanistically, it is known that anaphylatoxins are associated with cytokine induction and immune cell recruitment [22] and that TCC deposition mediates the induction of catabolic enzyme expression, inflammatory response, and regulated cell death of chondrocytes [20,21]. Considering previous findings in osteoarthritic cartilage, we assume that complement activation and subsequent promotion of pro-inflammatory processes may similarly play a pathobiological role in DD [16].

With the aim to address complement-associated dysfunctions and related diseases, several therapeutic approaches were developed to specifically inhibit the complement cascade. Cp40, a compstatin analog which is in clinical trial for periodontitis treatment, effectively inhibits activation of the central complement factor C3 (Figure 1) [23,24]. Eculizumab, a monoclonal antibody against complement factor C5, is in clinical use for the treatment of atypical hemolytic–uremic syndrome (aHUS) [25] and the hemolytic disease paroxysmal nocturnal hemoglobinuria (PNH) [26]. The antibody inhibits cleavage of C5 into the anaphylatoxin C5a and C5b, the initial component of TCC assembly, thereby blocking the terminal pathway of the complement (Figure 1) [26].

In this study, we investigated for the first time the cellular consequences of terminal complement activation, including anaphylatoxin production and TCC deposition, as a potential therapeutic target in the context of DD, and we examined its inhibitory effects on C3 and C5 levels. Furthermore, we analyzed the possible contribution of soluble factors released by degenerated EP tissue in direct complement activation on the C5 level.

## 2. Materials and Methods

### 2.1. Sample Collection

IVD tissue biopsies were obtained from 26 patients (16 women/10 men, 63.5 ± 19.5 years old) with disc degeneration (DD) undergoing surgery in the lumbar region. The study was conducted in accordance with the Declaration of Helsinki and approved by the local Ethics Committee of Ulm University, Germany (approval code 208/15) on the 9th of July 2015 and the Ethics Committee of Heidelberg University, Germany (approval code S-051/2016) on the 12th of February 2016.

### 2.2. Sample Processing

IVD tissue samples were macroscopically separated into annulus fibrosus (AF), nucleus pulposus (NP), and endplate (EP), and IVD cells were enzymatically isolated as previously described [19]. Isolated IVD cells were seeded at a density of 3000 cells/cm^2^ in IVD medium (DMEM with low glucose, 1% penicillin–streptomycin (Gibco, New York, NY, USA), 0.5% amphotericin B (Biochrom, Berlin, Germany), 1% non-essential amino acids (Biochrom, Berlin, Germany), and 1.5% 5 M NaCl/0.4 M KCl solution, as described in [19]) with 10% heat-inactivated fetal bovine serum (FBS, Gibco, New York, NY, USA) and cultured at 37 °C with 5% CO_2_. At a confluency of 70–80%, cells were detached via incubation with 0.05% trypsin–EDTA (Gibco, New York, NY, USA) for 10 min at 37 °C and expanded up to passages 2–4.

### 2.3. IVD Cell Culture Stimulations

For cell culture stimulation, isolated IVD cells were stimulated in IVD medium with 10% human serum (HS, Quidel, San Diego, CA, USA) alone or with the addition of 100 µg/mL zymosan (CompTech, Tyler, TX, USA) or of 0.5 µg/mL cathepsin D (CTSD, R&D Systems, Minneapolis, MN, USA) and treated with 5 µM Cp40 (produced from the Department of Pathology and Laboratory Medicine, University of Pennsylvania, Philadelphia, PA, USA) or 1 µM eculizumab (Soloris^TM^, obtained from remnants in infusion pipes), respectively. Cells in serum-free IVD medium served as the control group.

### 2.4. Cell-Based TCC ELISA

To quantify TCC deposition on cultured and stimulated human IVD cells, a cell-based ELISA was used [27]. 6000 cells/well were seeded on a 96-well plate and stimulated for 2 h as described previously. After stimulation, cells were fixed with 4% formalin for 15 min, and TCC deposition was detected as follows. Briefly, after a blocking step with 5% bovine serum albumin (Sigma-Aldrich, Taufkirchen, Germany) in DPBS at 37 °C for 1 h, cells were incubated for 2 h at 37 °C with an anti-C5b-9/TCC antibody (1:4000 dilution, Abcam, Cambridge, UK) and subsequently with an anti-rabbit IgG HRP conjugated antibody (1:10,000 dilution, Sigma-Aldrich, Taufkirchen, Germany) for 1 h at room temperature. Afterward, 3,3′,5,5′-tetramethylbenzidine (TMB) substrate (Sigma-Aldrich, Taufkirchen, Germany) was added for 20 min at room temperature. Finally, substrate conversion was stopped with stop solution (R&D Systems, Minneapolis, MN, USA), and absorbance was determined at 450 nm (microplate reader, infinite M200 pro, Tecan, Crailsheim, Germany).

### 2.5. RNA Isolation and Quantitative Real-Time PCR (RT-qPCR)

For gene expression analyses, 100,000 AF cells/well were seeded on a 24-well plate and stimulated for 24 h as described previously. Subsequently, the mRNA of the cells was isolated using a RNeasy Mini Kit (Qiagen, Hilden, Germany). cDNA was reverse transcribed with Super Script II Reverse Transcriptase (Invitrogen, Carlsbad, CA, USA) and used for quantitative real-time PCR analysis (StepOne-PlusTM Real-Time PCR System; Applied Biosystems, Darmstadt, Germany). GAPDH was used as a housekeeping gene, and relative gene expression levels in relation to the gene expression levels of the untreated control group were analyzed using the ∆∆CT method [28]. The following TaqMan Gene Expression Assays were used: *ADAMTS4* (Hs00192708), *GAPDH* (Hs02758991), *MMP1* (Hs00899658), and *PTGS2/COX2* (Hs00153133). 

### 2.6. C3a ELISA and C5a ELISA

Concentrations of anaphylatoxins C3a and C5a in cell culture supernatants of AF cells (24 h HS exposure) were analyzed using human ELISA kits (Invitrogen, Carlsbad, CA, USA, BMS2089 and DRG diagnostics, Marburg, Germany, EIA-3327) according to the manufacturer’s instructions.

### 2.7. Quantification of MMP1 and PGE2 in Cell Culture Supernatants

The MMP1 as well as PGE2 content of cell culture supernatants (AF cells, 24 h culturing) were analyzed using a Human Total MMP-1 DuoSet ELISA (R&D Systems, Minneapolis, MN, USA, DY901B) and a PGE2 ELISA kit (Enzo lifesciences, New York, NY, USA, ADI-900-001) by following the manufacturer’s instructions. Stimulation medium incubated for 24 h at 37 °C without cell contact served as a blank. MMP1 and PGE2 blank values, respectively, were subtracted from sample values. 

### 2.8. C5a Assay

In accordance with Ignatius et al. [29], 6000 AF cells/well were seeded in a 96-well plate and incubated with 20 µg/mL C5 (Quidel, San Diego, CA, USA) in serum-free IVD medium for 4 h. To analyze if different mediators associated with DD modulated the C5 cleavage capacity of AF cells, 10 ng/mL tumor necrosis factor (TNF, Gibco, New York, NY, USA), 10 ng/mL interleukin-1ß (IL-1ß, R&D Systems, Minneapolis, MN, USA), or 0.5 µg/mL CTSD (R&D Systems, Minneapolis, MN, USA) were added to some cells, and other cells were cultured in EP-conditioned medium (EPCM, EP tissue of DD patients cultured in serum-free IVD medium for 24 h, as previously described [30], pooled from 3 different donors). After stimulation, C5a concentrations in supernatants were analyzed using a human C5a ELISA kit (DRG Diagnostics, Marburg, Germany, EIA-3327).

### 2.9. Immunohistochemical Staining of TCC (C5b-9) and CTSD 

For the immunohistochemical staining of IVD tissue, paraffin sections of 3.5 µm thickness were used. TCC deposition as well as CTSD in IVD tissue were examined via immunohistochemical staining using an Agilent LSAB2 System HRP kit (Dako, Hamburg, Germany). For antigen retrieval, sections were incubated with hyaluronidase (2 mg/mL in 10 mM citrate buffer, pH 8, 30 min, 37 °C). TCC deposition was detected with a mouse anti-human C5b-9/TCC antibody (1:250, Quidel, San Diego, CA, USA, incubating overnight at 4 °C). CTSD deposition was detected with a mouse anti-human CTSD antibody (1:200, Abcam, Cambridge, UK, incubating overnight at 4 °C). Cell nuclei staining was performed with hematoxylin according to Mayer. Stained sections were imaged with light microscopy (Zeiss, Oberkochen, Germany).

### 2.10. Statistical Analysis

The results are presented in box-and-whisker plots as median ± interquartile ranges, showing all points. Normality was confirmed by using the Shapiro–Wilk normality test, and statistical analysis was performed with the parametric one-way ANOVA, followed by Sidak’s multiple comparison test, using GraphPad Prism 9 (GraphPad Software, Inc., La Jolla, CA, USA). Statistical significance was considered for *p* < 0.05.

## 3. Results

### 3.1. Zymosan Induces Enhanced TCC Deposition on IVD Cells

**Figure 2:**
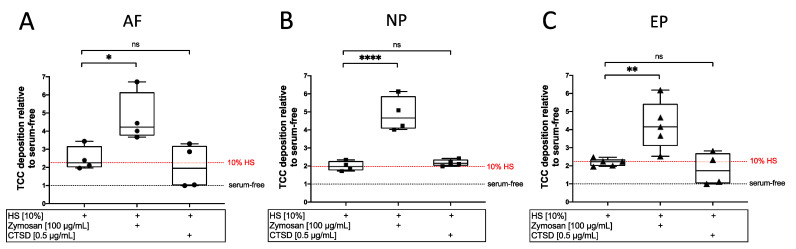
Evaluation of complement activation and subsequent TCC deposition induced by zymosan or cathepsin D (CTSD). Quantification of TCC deposition on isolated (**A**) annulus fibrosus (AF), (**B**) nucleus pulposus (NP), and (**C**) endplate (EP) cells stimulated with 10% human serum (HS) alone or with zymosan (100 µg/mL) or CTSD (0.5 µg/mL) for 2 h. Respective cells cultured in serum-free medium served as the control group. Sample values were normalized by the respective values of the serum-free control group (marked with black dotted line). Median TCC deposition after stimulation with 10% HS is indicated as a red dotted line. The results are presented as box-and-whisker plots with median ± interquartile ranges (*n* = 4–5 donors per group). Significant differences are depicted as: * *p* < 0.05; ** *p* < 0.01; **** *p* < 0.0001; ns: not significant. One-way ANOVA was used.

To establish a model of effective complement activation, IVD cells were cultured in IVD medium containing 10% HS and stimulated with either zymosan, a known activator of the alternative pathway [31], or CTSD, which is described as a direct activator of the terminal pathway via cleavage of C5 under serum-free conditions [32]. The potency of the stimulants concerning complement activation and TCC formation was quantified via a cell-based ELISA. Zymosan significantly increased the relative TCC deposition up to over fourfold compared to an around twofold deposition for 10% HS alone ((HS vs. HS + Zymosan): AF: *p* < 0.05; NP: *p* < 0.0001; EP: *p* < 0.01; Figure 2). However, CTSD did not further increase TCC deposition when compared to 10% HS. Therefore, zymosan was selected as complement activator for subsequent experiments.

### 3.2. Complement Inhibition by Either Cp40 or Eculizumab Decreases TCC Deposition on IVD Cells

**Figure 3:**
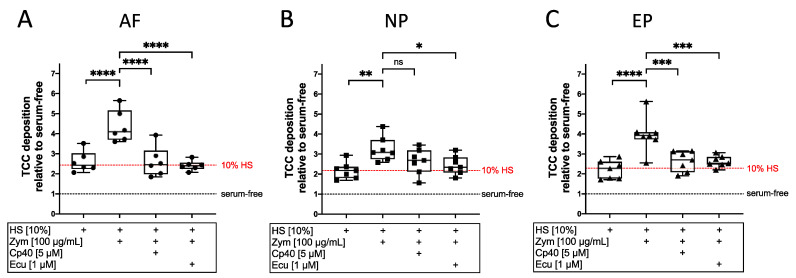
Investigation of complement inhibition regarding TCC deposition. TCC deposition on isolated (**A**) annulus fibrosus (AF), (**B**) nucleus pulposus (NP), and (**C**) endplate (EP) cells after 2 h stimulation with 10% human serum (HS) alone or with zymosan (100 µg/mL; Zym) with or without complement inhibitor Cp40 (5 µM) or eculizumab (1 µM; Ecu) analyzed via cell-based ELISA. Serum-free cell cultures served as the control group. Values of samples were normalized by respective values of the control group. Dotted lines represent the median TCC deposition of serum-free controls (black) and 10% HS (red). The results are presented as box-and-whisker plots with median ± interquartile ranges (*n* = 6–7 donors per group). Significant differences are depicted as: * *p* < 0.05; ** *p* < 0.01; *** *p* < 0.001; **** *p* < 0.0001; ns: not significant. One-way ANOVA was used.

Effective concentrations of the inhibitors were tested in a dose-finding study based on the quantification of TCC deposition on isolated AF cells (Cp40: 1, 2.8, and 5 µM; eculizumab: 0.5, 1, and 5 µM; Supplementary Materials Figure S1). To investigate the activation and inhibition of the complement cascade, isolated IVD cells were stimulated with HS and zymosan in the presence or absence of the complement inhibitors Cp40 (5 µM; inhibiting on the C3 level) or eculizumab (1 µM; inhibiting on the C5 level). After stimulation, TCC deposition was quantified using a cell-based ELISA. In the presence of 10% HS, relative TCC deposition was significantly increased on AF (*p* < 0.0001, 2.9-fold increase (vs. HS)), NP (*p* < 0.01, 1.4-fold increase (vs. HS)), and EP cells (*p* < 0.0001, 1.7-fold increase (vs. HS)) by stimulation with zymosan (Figure 3). The addition of Cp40 significantly reduced the TCC deposition on AF (*p* < 0.0001, reduction by 40%) and EP cells (*p* < 0.001, reduction by 30%) compared to stimulation with 10% HS and zymosan. Complement inhibition with eculizumab significantly reduced the zymosan-mediated increase of TCC deposition to the level of stimulation with 10% HS alone ((10% HS + zymosan vs. 10% HS + zymosan + eculizumab): AF: *p* < 0.0001; NP: *p* < 0.05; EP: *p* < 0.001).

### 3.3. Complement Inhibition in AF Cell Culture Suppresses Anaphylatoxin Generation

**Figure 4:**
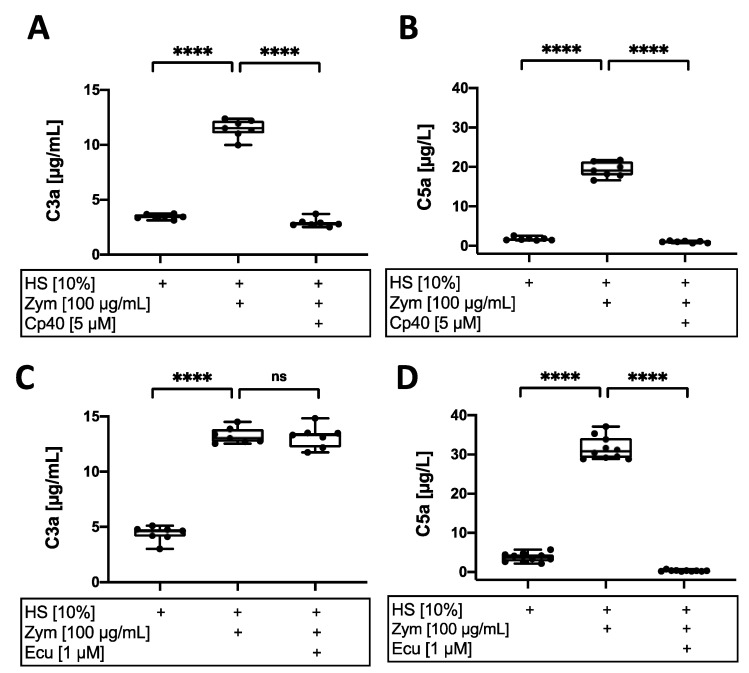
Effect of complement inhibition on anaphylatoxin generation. Analysis of (**A**,**C**) C3a (µg/mL) and (**B**,**D**) C5a concentrations (µg/L) in annulus fibrosus (AF) cell culture supernatants after 24 h of stimulation with 10% human serum (HS) alone or together with zymosan (100 µg/mL; Zym) with or without supplementation of (**A**,**B**) Cp40 (5 µM) or (**C**,**D**) eculizumab (1 µM; Ecu). The results are presented as box-and-whisker plots with median ± interquartile ranges (*n* = 6–10 donors per group). Significant differences are depicted as: **** *p* < 0.0001; ns: not significant. One-way ANOVA was used.

Complement activation after stimulation with zymosan with or without complement inhibitors was further analyzed via quantification of anaphylatoxins C3a and C5a in the supernatant of AF cell cultures with ELISA. C3a generation was about threefold higher in presence of zymosan than in cell culture supernatants with 10% HS alone (*p* < 0.0001), whereas C5a generation was increased >8-fold (*p* < 0.0001; Figure 4). The addition of the C5 antibody eculizumab inhibited C5a generation by 99% (*p* < 0.0001), whereas C3a generation was unaffected. In contrast, the addition of the C3 inhibitor Cp40 significantly decreased both C3a generation by 76% (*p* < 0.0001) and C5a generation by 95% (*p* < 0.0001) compared to stimulation with zymosan and HS. Taken together, both Cp40 as well as eculizumab efficiently inhibited C5a generation and TCC deposition; however, the generation of C3a was only inhibited by Cp40.

### 3.4. Complement Activation Induces Expression of Catabolic Enzymes in AF Cells

**Figure 5:**
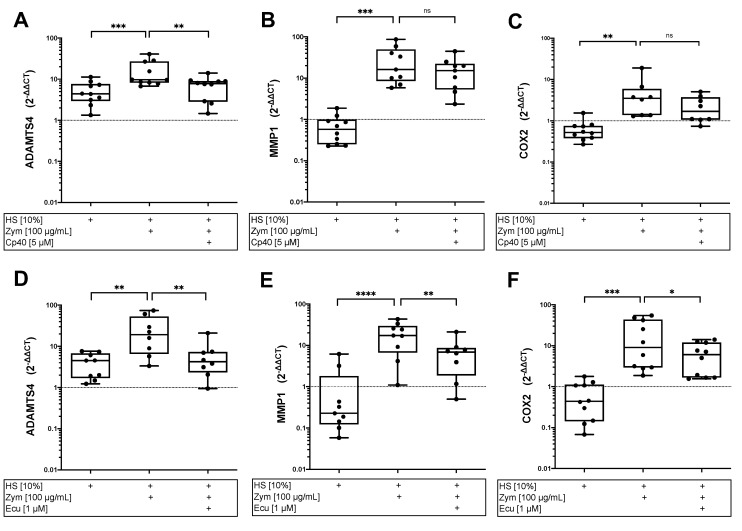
Effect of complement activation and inhibition on gene expression of annulus fibrosus (AF) cells. Gene expression analysis of catabolic enzymes ADAMTS4 (**A**,**D**) and MMP1 (**B**,**E**) as well as inflammation marker COX2 (**C**,**F**) in AF cells stimulated for 24 h with 10% human serum (HS) alone or with zymosan (Zym, 100 µg/mL) and treated with complement inhibitors Cp40 (5 µM; (**A**–**C**)) and eculizumab (Ecu, 1 µM; (**D**–**F**)). Relative gene expressions are presented as box-and-whisker plots with median ± interquartile ranges (*n* = 8–10 donors per group). Significant differences are depicted as: * *p* < 0.05; ** *p* < 0.01; *** *p* < 0.001; **** *p* < 0.0001; ns: not significant. One-way ANOVA was used.

To investigate the cellular response of complement activation, gene expression analyses of AF cells exposed to HS were performed. Stimulation with zymosan and HS significantly induced the gene expression of the catabolic enzymes ADAMTS4 and MMP1 and inflammation marker COX2 compared to HS alone (Figure 5). Treatment with eculizumab significantly reduced this induction for ADAMTS4 (*p* < 0.01), MMP1 (*p* < 0.01), and COX2 gene expression (*p* < 0.05) but could not completely inhibit it. Inhibition on the C3 level with Cp40 also suppressed the gene expression of ADAMTS4 (*p* < 0.01).

**Figure 6:**
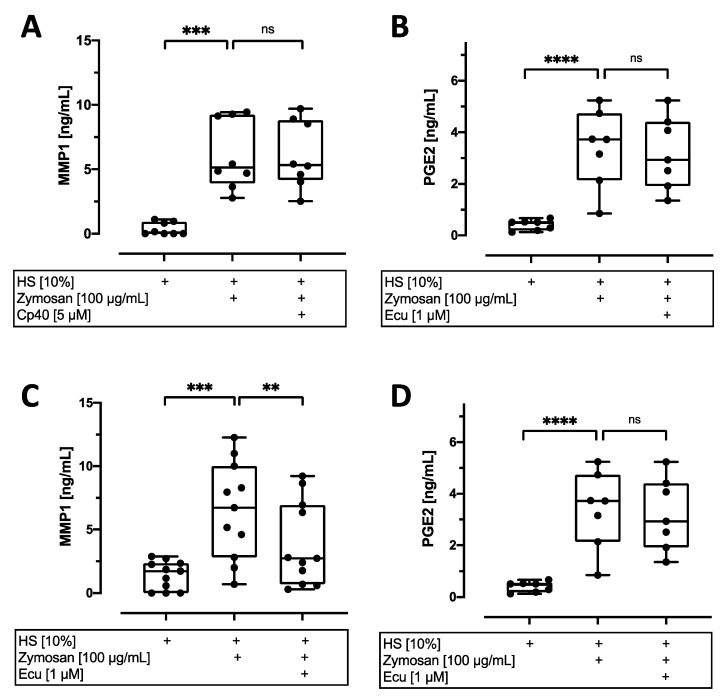
Quantification of MMP1 and PGE2 release from annulus fibrosus (AF) cells. Analysis of MMP1 (**A**,**C**) and PGE2 (**B**,**D**) concentrations in cell culture supernatants of AF cells after 24 h of stimulation with 10% human serum (HS) with or without zymosan (100 µg/mL) and treatment with 5 µM Cp40 (**A**,**B**) or 1 µM eculizumab (Ecu, (**C**,**D**)). The results are presented as box-and-whisker plots with median ± interquartile ranges (*n* = 8–11 donors per group). Significant differences are depicted as: ** *p* < 0.01; *** *p* < 0.001; **** *p* < 0.0001; ns: not significant. One-way ANOVA was used.

Subsequent analysis of MMP1 release was in accordance with the observations made on gene expression level. Stimulation with HS and zymosan significantly increased MMP1 release ((vs. HS): *p* < 0.001). This induction was reduced by eculizumab (−60%, *p* < 0.01), whereas C3 inhibition revealed no significant effect on MMP1 release (Figure 6A,C). The detection of PGE2 as a downstream product of COX2 could only partly reflect the effect observed for COX2 gene expression. The addition of zymosan significantly induced PGE2 release by AF cells exposed to 10% HS (by around a sevenfold increase, *p* < 0.0001; Figure 6B,D). Inhibition of complement by Cp40 and eculizumab reduced PGE2 release by about 20% each (not significant).

### 3.5. Addition of EPCM or CTSD Can Modulate C5a Generation in AF Cell Cultures

In order to study whether AF cells alone or in presence of catabolic or pro-inflammatory mediators are able to cleave C5 and thereby contribute to complement activation, AF cells were cultured under serum-free conditions supplemented with C5 protein. ELISA-based quantification of C5a in cell culture supernatants was performed to detect subsequent C5 cleavage, in which C5a and C5b, the initial components of TCC formation, were formed in a stoichiometric ratio.

**Figure 7:**
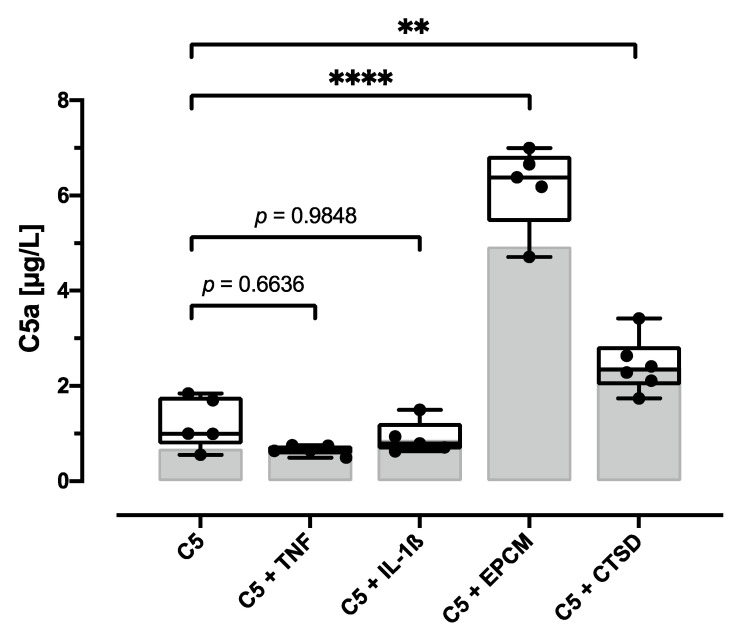
Analysis of C5 cleavage capacity. Quantification of C5a content in annulus fibrosus (AF) cell culture supernatants after 4 h cultivation in serum-free medium containing 20 µg/mL C5 only (C5) or supplemented with 10 ng/mL TNF (C5 + TNF), 10 ng/mL IL-1ß (C5 + IL-1ß), and 0.5 µg/mL CTSD (C5 + CTSD) as well as endplate-conditioned medium containing 20 µg/mL C5 (C5 + EPCM). The results are presented as box-and-whisker plots with median ± interquartile ranges (*n* = 5–6 donors per group). Grey bars represent the median C5a concentration of stimulation medium incubated for 4 h without cell contact (*n* = 2). Significant differences are depicted as: ** *p* < 0.01; **** *p* < 0.0001. One-way ANOVA was used.

Direct cleavage of supplemented C5 was not detected in AF cell cultures without additional catabolic or pro-inflammatory stimuli (Figure 7). Stimulation with the cytokines IL-1ß and TNF, which are considered to be key factors in IVD degeneration [33], did not influence C5 cleavage ((vs. C5), not significant). However, in supernatants of cultured AF cells in presence of CTSD, we observed enhanced C5a levels ((vs. C5), *p* < 0.01). Furthermore, C5a generation was increased threefold in the supernatants of AF cells stimulated with EPCM ((vs. C5), *p* < 0.0001). However, no C5a could be detected in the EPCM used per se (data not shown).

**Figure 8:**
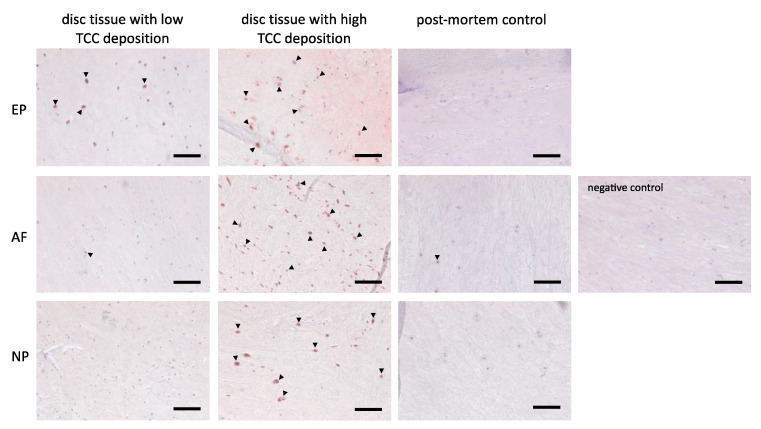
Immunohistochemical staining of cathepsin D (CTSD). Representative images of immune histological staining of CTSD in degenerated IVD tissue with remarkably high respective low TCC deposition, as well as macroscopically non-degenerated IVD tissue of a control donor collected post-mortem (60 years old, female) in the three different IVD regions (endplate (EP), annulus fibrosus (AF), and nucleus pulposus (NP)) and a representative negative control of the staining. CTSD-positive cells are indicated by arrowheads as examples. Scale bar: 100 µm.

To investigate if the presence of CTSD in degenerated IVD tissue can be associated with enhanced TCC deposition, exemplary immunohistochemical staining of CTSD was performed in IVD tissue obtained from DD patients. Based on previous investigations [18], tissue samples were thereby divided into samples with remarkably high vs. low TCC deposition levels. In samples with strong TCC deposition, higher levels of CTSD-positive cells were observed, whereas samples with lower numbers of TCC-positive cells exhibited less CTSD (Figure 8). In macroscopically non-degenerated control samples, TCC deposition and CTSD were detected only in low levels.

## 4. Discussion

Previous observations of TCC deposition in clinical IVD samples correlating with the grade of tissue degeneration imply that complement activation might be involved in the pathogenesis of DD [18]. To further study the role of complement activation in the pathogenesis of DD, we analyzed the cellular consequences of anaphylatoxin generation and TCC formation in isolated AF cells and investigated the inhibitory effects of Cp40 and eculizumab. In AF cells exposed to HS and zymosan, we identified a significant induction of ADAMTS4, MMP1, and COX2-mediators known to be involved in DD [3,8,11]. Furthermore, we showed that direct C5 cleavage is possible in AF cell cultures in the presence of EPCM, indicating that IVD cells might contribute to direct activation of the terminal complement cascade.

Based on the comparative evaluation of two different mediators of complement induction tested on isolated IVD cells, we chose zymosan as a commonly used complement activator [31,34]. In former studies, stimulation with zymosan did not increase TCC deposition on isolated IVD cells exposed to 5% HS. However, in IVD tissue cultures in which IVD cells were embedded in their natural microenvironment, a significant induction of TCC deposition mediated by zymosan was observed [19]. These differences may be explained by the cell-protective effect of membrane bound complement regulator proteins (CRegs) CD46, CD55, and CD59 (protectin), which are known to be strongly expressed in cultured IVD cells [19]. However, by increasing the serum concentration to 10% in the present study, we could show an enhanced TCC deposition on cultured IVD cells due to stimulation with zymosan, indicating that an increase in complement factors can overcome this self-protecting effect in isolated IVD cells.

The general effects of complement activation and inhibition on complement activation products (TCC depositions and anaphylatoxin generation) observed in the presence of IVD cells were in line with previously described specifications of Cp40 and eculizumab [26,35]. Therefore, the established in vitro model proved to be suitable for the analysis of the cellular effects of complement activation in AF cells.

Focusing on the cellular consequences of complement activation in IVD cells, we found that stimulation with zymosan and HS enhanced the gene expression levels of the catabolic enzymes ADAMTS4 and MMP1 as well as those of the inflammation marker COX2 in AF cells, which were significantly reduced via complement inhibition using eculizumab. These results were confirmed at the protein level for MMP1 and in trend for COX2 (downstream product PGE2).

Overall, our findings suggest that the impact of complement inhibition at C3 level (via Cp40, resulting in the significant inhibition of C3a, C5a, and TCC) was less effective concerning the expression of selected target genes than inhibition at C5 level (via eculizumab, resulting in the inhibition of C5a and TCC). Possibly, a difference in C3a generation could be somehow involved in this observation. Nevertheless, the inhibitory effect of Cp40 on C3a generation might have important therapeutic relevance in vivo with respect to other pathophysiological aspects of DD, such as the recruitment of immune cells.

Altogether, the observed induction of catabolic factors in AF cells exposed to HS and zymosan provides new insights into the potential influence of complement activation products on degenerative processes in IVD tissue [3,8,11]. In the context of OA, the TCC-mediated induction of ADAMTS4, MMP1, and COX2 was also described in isolated murine chondrocytes [20]. These catabolic factors are known to be involved in matrix degradation, especially the pathophysiological cleavage of aggrecan and collagen [36], and the decreased biosynthesis of proteoglycans [37]. In a human cartilage ex vivo model, complement activation was further associated with an increase of trauma-induced secretion of catabolic enzymes, such as MMP13, and inflammatory cytokines, such as IL-6, as well as phenotypical alterations (hypertrophy and senescence) of chondrocytes [21]. In consideration of the findings in OA mentioned above, the complement-mediated induction of catabolic enzymes and inflammation markers, as demonstrated in isolated AF cells, might similarly contribute to progressive tissue degeneration and inflammation in DD. 

In addition to conventional complement activation via the classical, lectin, or alternative pathways, as exemplified by zymosan, direct C5 cleavage by different factors, such as thrombin and CTSD, has been reported [32,38]. To study the potential relevance of direct C5 cleavage and subsequent terminal complement activation in human IVDs, a respective assay using AF cells was performed. With this approach, we could create a first proof that direct C5 cleavage is possible in AF cell cultures under degeneration-associated conditions and that it might occur in the context of DD. Isolated AF cells alone could not cleave C5, but the direct cleavage of C5 was observed in approaches in which AF cells were stimulated with EPCM or CTSD in the absence of HS. Although proteomic analyses of the EPCM have not been performed, it is very likely that EPCM contains various soluble mediators released by the degenerated tissue, such as cytokines, MMPs, or other proteases such as CTSD [7,9,10,30]. The lysosomal protease CTSD was previously identified as a matrix-degrading enzyme present in degenerated human IVD tissue [9], and therefore, it is one candidate to be implicated. In fact, the exemplary immunohistochemical staining of degenerated IVD tissue provided first evidence for an association between TCC deposition and the presence of CTSD. The CTSD-positive cells could partly represent immigrated immune cells, which should be clarified in future work. Interestingly, stimulation with CTSD in IVD cell cultures exposed to 10% HS did not induce TCC deposition, as determined via TCC ELISA. This striking difference to the C5 cleavage capacity observed under serum-free conditions may be explained by the presence of CTSD inhibitors in the serum, e.g., alpha-2-macroglobulin [39].

CTSD plasma concentration was found to be increased after severe tissue trauma, and it enables complement activation via direct cleavage of C5 [32]. In the cartilage and synovial fluids of OA patients, CTSD was detected in increased concentrations [40,41], which is primarily associated with matrix degeneration due to its proteolytic activity, but it may also be related to C5 cleavage [42]. We hypothesize that the degenerative environment in DD, CTSD expression in particular, may induce C5 cleavage and thereby activate the terminal complement cascade that contributes to TCC deposition in IVD tissue. A possible cleavage of C3 by components present in EPCM has not been investigated, but it should be addressed in future studies.

Besides TCC formation, C5 cleavage results in anaphylatoxin C5a generation, which strongly contributes to inflammation. C5a is involved in the recruitment of inflammatory cells, such as neutrophiles or monocytes, and it triggers the production of pro-inflammatory cytokines [43]. In OA pathogenesis, C5 activation plays a central role in the development of low-grade inflammation, which is associated with pain and progression of the disease [44]. C5a concentrations in the synovial fluid of patients suffering from rheumatoid arthritis were even higher than in the synovial fluid of OA patients [45], which reinforces that C5 activation is strongly associated with inflammation. Considering the infiltration of immune cells in degenerated IVDs [10,13] and the potential activation of C5, it might be possible that C5a contributes to inflammation in DD. As both complement inhibitors Cp40 and eculizumab effectively prevented C5a generation in our in vitro study, we could not differentiate between TCC- and C5a-mediated effects. The direct effects of anaphylatoxins, especially C5a, on IVD cells as well as of the influence of anaphylatoxins on immune cell recruitment are not included in this study but should be addressed in future studies to clarify the complement-associated mechanisms in DD pathology in more detail.

## 5. Conclusions

This study demonstrates that terminal complement activation is involved in the upregulation of MMP1, ADAMTS4, and COX2 in human IVD cells as mediators which are associated with IVD tissue degeneration. Inhibition on the C5 level reduced this upregulation, indicating that C5a and/or TCC deposition are involved in the induction of these catabolic factors. Soluble components secreted by degenerated EP tissue, such as CTSD, were observed to induce direct C5 cleavage and thereby might activate the terminal complement cascade, finally leading to C5a generation and TCC deposition. Therefore, we hypothesize that the process of terminal complement activation might be functionally involved in the pathogenesis of DD. Future studies should address the question of whether C5a generation, TCC deposition, or both are relevant for the development of specific therapeutic approaches to attenuate or prevent DD progression.

## Data Availability

Data are contained within the article.

## Supplementary Materials

The following supporting information can be downloaded at: https://www.mdpi.com/article/10.3390/cells12060887/s1, Figure S1: Dose-finding study of complement inhibitors based on evaluation of TCC deposition on AF cells.
