# Supplementary material for: Terminal Complement Activation Is Induced by Factors Released from Endplate Tissue of Disc Degeneration Patients and Stimulates Expression of Catabolic Enzymes in Annulus Fibrosus Cells
Source: Cells. 2023 Mar 13;12(6):887. doi: 10.3390/cells12060887 (PMC10047197; doi:10.3390/cells12060887)
Supplement: Supplementary file 1 [file cells-12-00887-s001.zip › cells-2264407-supplementary.pdf]

Supplementary Material

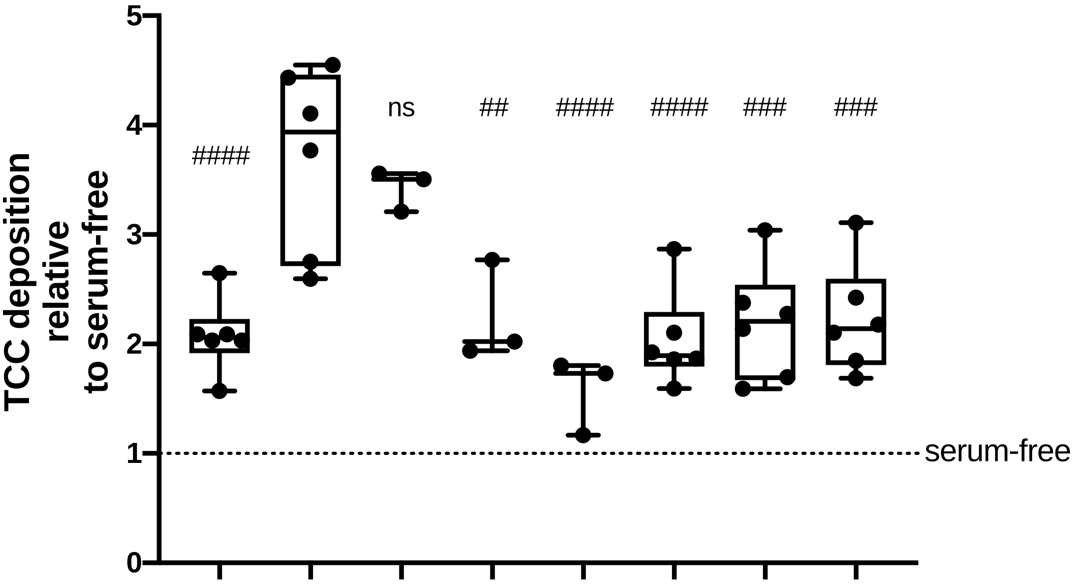

Human Serum [10%]	+	+	+	+	+	+	+	+
Zymosan [100 µg/mL]		+	+	+	+	+	+	+
Cp40 [µM]			1	2.8	5			
Eculizumab [µM]						0.5	1	5

Figure S1. Dose-finding study of complement inhibitors based on evaluation of TCC deposition on AF cells. Quantification of TCC deposition on isolated AF cells stimulated with 10% human serum (HS) alone or with zymosan (100 µg/mL) for 2 h. To determine the necessary concentrations of complement inhibitors used in cell culture experiments, AF cells stimulated with HS and zymosan were treated with C3 inhibitor Cp40 (1, 2.8 or 5 µM) or C5 inhibitor eculizumab (0.5, 1 or 5 µM). Respective cells cultured in serum-free medium served as control. Sample values were normalized by respective value of the serum-free control (marked with black dotted line). In case of Cp40, a concentration of 5 µM resulted in the strongest inhibition of TCC deposition and therefore was chosen as concentration used for subsequent experiments. Since all three concentrations of eculizumab tested resulted in comparable inhibitions of TCC deposition on AF cells, the median concentration of 1 µM was used in the following experiments. Results are presented as box-and-whisker plots with median ± interquartile range (n = 3-6 donors per group). Significant differences vs [HS + zymosan] are depicted as: $^{\#\#}P < 0.01$; $^{\#\#\#}P < 0.001$; $^{\#\#\#\#}P < 0.0001$; ns: not significant, one-way ANOVA.
